# Differences in Fibromyalgia Characteristics by Mode of Commute and Age in Women: The Al-Ándalus Project

**DOI:** 10.3390/healthcare12212168

**Published:** 2024-10-31

**Authors:** Manuel Herrador-Colmenero, Milkana Borges-Cosic, Víctor Segura-Jiménez, Inmaculada C. Álvarez-Gallardo, Palma Chillón, Manuel Delgado-Fernández

**Affiliations:** 1 La Inmaculada Teacher Training Centre, Sport and Health University Research Institute (IMUDS), University of Granada, 18012 Granada, Spain; 2 PA-HELP “Physical Activity for HEaLth Promotion” Research Group, University of Granada, 18013 Granada, Spain; milkana.borges@uca.es; 3 Department of Physical Education, Faculty of Education Sciences, University of Cádiz, 11003 Cádiz, Spain; 4 UGC Medicina Física y Rehabilitación, Hospital Universitario Virgen de las Nieves, 18014 Granada, Spain; vsegura@ibsgranada.es; 5 Instituto de Investigación Biosanitaria ibs.GRANADA, 18012 Granada, Spain; 6 GALENO Research Group, Department of Physical Education, Faculty of Education Sciences, University of Cádiz, 11519 Puerto Real, Spain; inma.alvarez@uca.es; 7 Biomedical Research and Innovation Institute of Cádiz (INiBICA) Research Unit, Puerta del Mar University Hospital, 11009 Cádiz, Spain; 8 Department of Physical Education and Sports, Faculty of Sport Sciences, Sport and Health University Research Institute (IMUDS), University of Granada, 18011 Granada, Spain; pchillon@ugr.es; 9 PA-HELP “Physical Activity for Health Promotion, CTS-1018” research group, Department of Physical Education and Sports, Faculty of Sports Science, University of Granada, 18011 Granada, Spain; manueldf@ugr.es; 10 Sport and Health University Research Institute (IMUDS), University of Granada, 18007 Granada, Spain

**Keywords:** exercise, active travel, chronic disease, female

## Abstract

Objectives: This study aimed to test whether fibromyalgia-related characteristics differ by mode of commute and by age in women with fibromyalgia. Methods: A total of 450 women with fibromyalgia (aged 52.0 ± 8.0 years old) were included. Data on their body composition, socioeconomic factors, health-related quality of life (HRQoL), depressive symptoms, sleep quality, pain-related outcomes, fatigue, and mode of commute, as well as the impact of fibromyalgia on their lives, were obtained. We performed linear regression analyses to test the differences in fibromyalgia-related characteristics between the participants engaged in active/passive modes of commuting. To analyze in depth the differences, a one-way analysis of covariance with Bonferroni’s correction for multiple comparisons was conducted. Results: In the younger group, active commuters presented differences in fibromyalgia-related characteristics (all, *p* < 0.01): they were less impacted by having fibromyalgia, had lower levels of fatigue, and had a higher HRQoL than passive commuters. We observed no differences in symptoms between active and passive commuters in the older group (all, *p* > 0.05). Conclusions: The possible positive effect of active commuting on fibromyalgia-related characteristics might be reduced by age and by a decrease in total physical activity. Physical activity programs for women with fibromyalgia focused on improving fibromyalgia-related characteristics should consider active commuting behaviors to increase their effectiveness.

## 1. Introduction

Fibromyalgia is a multi-symptomatic disorder of unknown etiology, related to alterations in pain modulation in the central nervous system [1]. The symptoms usually reported by fibromyalgia patients are altered sleep patterns, fatigue, cognitive difficulties, reduced functional abilities, and depression [2,3]. Fibromyalgia patients show a more impaired health status compared with patients with other pain conditions [3].

Participating in physical activity (PA) is associated with lower levels of pain, depression, and fatigue [4,5,6]; a lesser impact on one’s life; and a greater health-related quality of life (HRQoL) in fibromyalgia patients [4]. Moreover, a sedentary lifestyle contributes to reduced sleep quality among fibromyalgia patients [7] and a worse HRQoL [8]. The prevalence of sleep disorders in women with fibromyalgia has been reported to be above 90% and is associated with worse symptomatology [9]. Thus, an increase in PA and a reduction in sedentary time could improve these patients’ sleep quality and might reduce the severity of the disease [10]. Walking for commuting purposes (e.g., going to the supermarket) is an opportunity to increase one’s PA and to reduce one’s sedentary time [11], which might help in the management of fibromyalgia symptomatology. The health-related benefits that walking for commuting purposes might confer to the general population have been previously described [12]. Nevertheless, it is unknown whether these benefits might be generalizable to fibromyalgia patients. It has been shown that increasing the walking around 1000 steps/day in patients with fibromyalgia was associated with lower levels of physical impairment, pain interference, and depressive symptoms, as well as with a higher score in the standardized SF-36 physical component summary [13]. A recent study in women with fibromyalgia evidenced that unsupervised but structured walking was associated with lower levels of perceived fatigue [6]. Additionally, the severity of symptoms such as anxiety and tenderness, their perceptions of intense pain, and their perceptions of insomnia decreased after walking 1.25 km in natural environments, while their perceptions of their own well-being increased [14]. However, some patients perceived walking as a challenging activity that might worsen their symptomatology [15,16], which might lead them to avoid this activity. There are not enough previous observational studies in the literature addressing both fibromyalgia and active commuting. Previous results have revealed that age modifies the mode of commute in women with fibromyalgia compared with the general population [17], showing lower percentages of active commuting among women with fibromyalgia aged <51 years old compared to their general counterparts. This age effect in the mode of commute observed in women with fibromyalgia has also been reported in healthy adults [18]. It is of interest to corroborate the association of active commuting and fibromyalgia symptomatology in a large sample in order to design experimental research to address the causality of these findings.

Fibromyalgia places a considerable burden on patients (e.g., a reduced quality of life) [2,19] and society (e.g., an increased use of health services) [20]. Currently, there is no universal treatment to help patients efficiently manage fibromyalgia [21], probably because of the high heterogeneity of the disease [22]. All patients are able to implement healthy behaviors in their daily routines, which might improve their symptomatology as well as reduce their use of health resources. However, it is unknown how active commuting is related to fibromyalgia characteristics, and this would constitute valuable information to consider when physical activity interventions are designed. Thus, this study aimed to test whether fibromyalgia-related characteristics differ by mode of commute and age in women with fibromyalgia. Additionally, to deepen our understanding of any differences, patients were divided into groups by mode of commute (i.e., active vs. passive) and age (i.e., younger vs. older) in our analysis. This study hypothesized that women with fibromyalgia who are younger and active commuters have fewer symptoms than women with fibromyalgia who are older and passive commuters.

## 2. Materials and Methods

### 2.1. Study Participants and Design

We contacted a total of 579 women with fibromyalgia from Southern Spain. The recruitment procedure of a representative sample of women with fibromyalgia for the current cross-sectional study has been previously described [2]. The inclusion criteria were as follows: (a) women who had been previously diagnosed with fibromyalgia by a rheumatologist; (b) who met the 1990 American College of Rheumatology Fibromyalgia criteria [23]; (c) who did not have severe or terminal illnesses or severe cognitive impairments (Mini Mental State Examination score ≤ 10) [24]; and (d) who fully completed the mode of commute questionnaire and supplied their symptomatology.

Data collection was conducted between 2011 and 2013. After receiving detailed information about the aims of the study and procedures involved and before taking part in the study, the participants gave their written informed consent. The participants attended a measurement session at which the inclusion criteria were confirmed. Moreover, their body compositions were measured, and their socioeconomic factors were self-reported on a questionnaire. Thereafter, the participants were instructed to complete the questionnaires. On a second day, the research team verified whether the questionnaires had been properly and completely filled in. This study was reviewed and approved by the Ethics Committee of the “Hospital Virgen de las Nieves” (Granada, Spain).

### 2.2. Variables

#### 2.2.1. Adiposity

To assess adiposity, body fat percentage was measured using a valid and reliable portable eight-electrode tactile bioelectrical impedance analysis device (InBody R20, Biospace, Seoul, Republic of Korea) [25,26].

#### 2.2.2. Socioeconomic Factors

The socioeconomic factors were assessed through a questionnaire that was adapted from the Spanish National Health survey 2006. Household members (i.e., whether the patient had others living at home or they lived alone), educational level (i.e., no formal education, primary school education, professional training, secondary school education, and/or university degree), and current occupational status (i.e., working/studying and unemployed/retired) were self-reported.

#### 2.2.3. Impact of Fibromyalgia

The impact of fibromyalgia on the patients was assessed with the Spanish version of the Revised Fibromyalgia Impact Questionnaire (FIQR), which is a valid tool to discriminate fibromyalgia patients from patient with other diseases [27]. In this self-administered questionnaire, each of the 21 items included a rating scale of 0 to 10 [27]. The FIQR total score (0–100) was calculated. Higher scores in this questionnaire indicate a greater effect of the condition on the person’s life.

#### 2.2.4. Health-Related Quality of Life

To assess the physical and mental components of the HRQoL, we used the Spanish version of the 36-item Short-Form Health Survey (SF-36) [28], which is a reliable (intra-class correlation = from 0.6 to 1.0) tool. The standardized physical and mental component summaries (with a range of 0–100) were used, in which higher scores represent better physical and mental HRQoL.

#### 2.2.5. Depressive Symptoms

The Spanish version of the Beck Depression Inventory—Second Edition (BDI-II) [29] was used to assess depressive symptoms. This reliable (intra-class correlation = 0.9) tool has 21 items and addresses symptoms in the previous 2 weeks. The participants rated each item on a 0–3 scale (0 = not present; 3 = severe). The overall score (with a range of 0–63) provided by the BDI-II is calculated, and scores of ≤13, 14–19, 20–28, and ≥29 represent minimal, mild, moderate, and severe depressive symptoms [30], respectively.

#### 2.2.6. Sleep Quality

The Spanish version of the Pittsburgh Sleep Quality Index (PSQI) [31] is a valid (adequate convergent validity with Fibromyalgia Impact Questionnaire and SF-36) and reliable (test/retest Spearman correlation from 0.4 to 0.7) tool and was used to assess the patients’ sleep quality in the current study. Total PSQI scores range from 0 to 21, in which higher scores represent worse sleep quality.

#### 2.2.7. Pain-Related Outcomes

Standard pressure algometry (FPK 20; Wagner Instruments, Greenwich, CT, USA) was used to measure experimental pain. Eighteen fibromyalgia-related tender points [23] were examined. The tender points described by Wolfe et al. [23] are occiput, low cervical, trapezius, supraspinatus, second rib, lateral epicondyle, gluteal, greater trochanter, and knee, in both right and left body sides. A pressure threshold of ≤4 kg/cm^2^ was defined as a positive tender point. Two alternative measurements at each tender site were taken. The mean score of tender points was recorded as pressure pain threshold.

Pain-related catastrophizing was assessed with the Spanish version of the Pain Catastrophizing Scale [32]. This valid and reliable (intra-class correlation = 0.8) scale [32] is a 13-item questionnaire in which women were asked to reflect on past painful experiences and indicate their thoughts or feelings about pain. Each item includes a 5-point scale. The total score (with a range of 0–52) was used, where higher scores represent a more negative appraisal of pain.

Efficacy expectations for coping with pain were assessed with the Spanish version of the Chronic Pain Self-Efficacy Scale [33]. This valid and reliable (Pearson correlation = from 0.5 to 0.9) questionnaire [33] contains 19 items grouped into a coping, function, and pain subscale. The total score was calculated by the sum of the three subscales (with a range of 0–300), in which a higher score was a better result.

#### 2.2.8. Fatigue

Fatigue severity was assessed with the Spanish version of the Multidimensional Fatigue Inventory (MFI-S) [34,35], which is a reliable (intra-class correlation = from 0.64 to 0.91) and validated tool (weak-to-fair significant positive relationship with global fatigue in the Fibromyalgia Impact Questionnaire). This questionnaire is composed of 5 scales: general fatigue, physical fatigue, reduced activity, reduced motivation, and mental fatigue. Four items comprise each subscale with five-point Likert scales. Only the general fatigue scale was used (with score range from 4 to 20), in which higher scores indicate greater fatigue.

#### 2.2.9. Mode of Commute

The mode of commute was assessed using the questions in the Spanish version of the Assessing Levels of Physical Activity environmental questionnaire, which is a valid (correlated with moderate PA measured with accelerometry) and reliable (intra-class correlation = 0.86) self-administered scale [36]. The questions on their mode of commute refer to four different destinations: local shops, supermarkets, local services, and school/work. For this study, the school/work item was not used due to the low number of participants who reported studying or working. From the response options in each question (i.e., walking, cycling, driving, riding a motorcycle, taking the bus/metro/train, and others), only one of them could be chosen. The three modes of commuting questions were recorded as active (i.e., walking and cycling) or passive (driving, riding a motorcycle, and taking the bus/metro/train). The option “other” was included as active or passive if participants specified the mode of transportation, but if they did not, this response was excluded from the analysis. Additionally, the variable for active commuting was created by the sum of the three destinations (i.e., commuting to local shops, commuting to supermarkets, and commuting to local services). Having no active responses or one active response was recoded as passive commuting, and having two or more active responses was recoded as active commuting.

### 2.3. Statistical Analysis

Since previous results have revealed that age modifies the mode of commute (active vs. passive) [17] among women with fibromyalgia between 35 and 65, the analyses were conducted separately in two age groups (ages < 51 and ≥51). To test differences in fibromyalgia-related characteristics between the younger and older group, we performed Student’s *t*-test for continuous variables (i.e., age, body fat percentage, fibromyalgia impact, HRQoL, depressive symptoms, sleep quality, pain-related outcomes, and fatigue variables) and chi-square for categorical variables (i.e., socioeconomic factors, active commuting, and modes of commute to local shops, supermarkets, and local services).

We performed linear regression analyses for the different age groups to test differences in fibromyalgia-related characteristics (main outcome) between the participants engaged in active and passive modes of commute (exposure variable). Among the fibromyalgia-related characteristics that showed differences across the mode of commute, we created a 4-category variable combining age group and mode of commute (i.e., where 0 = younger and active commuters; 1 = younger and passive commuters; 2 = older and active commuters; and 3 = older and passive commuters). One-way analysis of covariance with Bonferroni’s correction for multiple comparisons was conducted to assess the differences in each fibromyalgia-related characteristic (dependent variable) across categories of this new variable (independent variable). Despite other potential confounders (i.e., having other household members, educational level, and occupational status), we used age as a covariate for all analyses.

All analyses were undertaken using Stata v.13. The level of significance was set at *p* < 0.05.

## 3. Results

From the total of 579 participants who agreed to participate in the study, 92 women did not meet the 1990 ACR fibromyalgia criteria, one woman had severe cognitive dysfunction, and 36 women did not provide complete data on their mode of commute and fibromyalgia-related characteristics. A final sample of 450 women with fibromyalgia was included in the present study.

Table 1 shows the age, fibromyalgia-related characteristics, and mode of commute in the participants with fibromyalgia. In comparison with the older group, the younger group had a higher level of education (4.13% vs. 15.6%; *p* < 0.001), a higher occupational status (35% workers vs. 19.1% students; *p* < 0.001), a lower body fat percentage (38.1 vs. 40.9; *p* < 0.001), a higher chronic pain self-efficacy total score (142.3 vs. 130.6; *p* < 0.05), a higher MFI-S general fatigue score (18.3 vs. 17.7; *p* < 0.05), and reported actively commuting to supermarkets less frequently (41.3 vs. 50.8%, *p* < 0.05). However, there were no differences in terms of whether they had other members in their household, their pressure pain threshold scores, FIQR total scores, SF36 physical and mental scores, BDI-II total scores, PSQI total scores, pain-related catastrophizing total scores, and whether they actively commuted to local shops and local services (all, *p* > 0.05).

Table 2 shows the differences in fibromyalgia-related characteristics between active and passive commuters by age groups. In the younger group, active commuters presented a significantly lower FIQR total score (with a mean difference: 7.6; 95% confidence interval (95% CI): 2.4–12.8; *p* = 0.004), MFI-S general fatigue score (with a mean difference: 1.0; 95% CI: 0.3–1.7; *p* = 0.009), and higher SF36 physical score (with a mean difference: –3.6; 95% CI: −5.6–1.6; *p* < 0.001) than passive commuters. We observed no differences in symptoms between active and passive commuters in the older group (all, *p* > 0.05).

**Figure 1:**
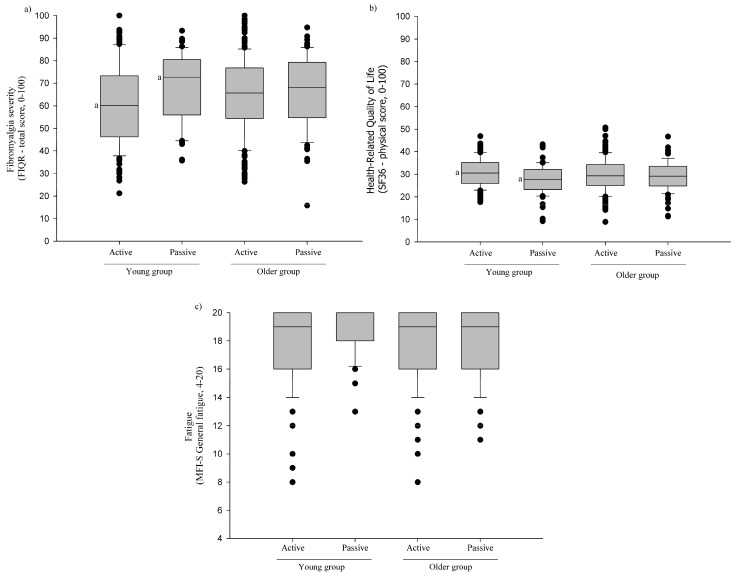
Differences (ANCOVA) in (**a**) Fibromyalgia Impact Questionnaire Revised (FIQR), (**b**) 36-item Short-Form Health Survey (SF-36) Physical Score, and (**c**) Spanish version of the Multidimensional Fatigue Inventory (MFI-S) general fatigue scores among groups by age combined with mode of commute (i.e., young and active commuters; young and passive commuters; old and active commuters; and old and passive commuters), adjusted for age. Common superscripts indicate significant (*p* < 0.05) differences between the groups with the same letter after Bonferroni’s correction.

Figure 1 illustrates the differences in FIQR total scores, SF36 physical scores, and MFI-S general fatigue scores between the groups by age and mode of commute. Young and active commuters showed lower FIQR total scores than young and passive commuters (with a mean score of 59.3 and standard deviation (SD): 1.9 units vs. a mean score of 67.0 and SD: 2.6 units, respectively; *p* < 0.05). The results of young and active commuters in terms of their FIQR total scores (with a mean score 59.3, SD: 1.9 units) and the results of old and passive commuters (with a mean score 67.5, SD: 2.1 units) differed, although this difference did not reach statistical significance (*p* = 0.065). Regarding their HRQoL, young and active commuters presented higher SF36 physical scores than young and passive commuters (with a mean score of 31.5 and SD: 0.8 units vs. a mean score of 28.0 and SD: 1.1 units; *p* < 0.01). Finally, we found no differences in MFI-S general fatigue between the groups by age and mode of commute (all, *p* > 0.05), although young and active commuters showed lower MFI-S general fatigue scores than young and passive commuters without significant statistical differences (a mean score of 17.5 and SD: 0.3 units vs. a mean score 18.4 and SD: 0.4 units; *p* = 0.09).

## 4. Discussion

This study hypothesized that women with fibromyalgia who are younger and active commuters have fewer symptoms than women with fibromyalgia who are older and passive commuters. Only among the young women with fibromyalgia, active commuters reported they were less impacted by fibromyalgia, had lower levels of fatigue, and had a higher HRQoL than passive commuters. When the groups divided by age and mode of commute (i.e., younger and active commuters, younger and passive commuters, older and active commuters, and older and passive commuters) were analyzed, the results remained consistent, except for the results for fatigue. Younger and active commuters were shown to be less impacted by fibromyalgia and had a higher HRQoL than their younger and passive counterparts, although the differences found were relatively small.

This study represents the first comprehensive characterization of the association between active commuting and fibromyalgia-related characteristics in women with fibromyalgia. We found different results depending on the age group in some fibromyalgia-related characteristics: the results suggest that active commuting might be related to the impact of fibromyalgia, patient HRQoL, and fatigue scores only in young women with fibromyalgia. Associations between active commuting and fibromyalgia-related characteristics vary with age, which might be due to differences in fibromyalgia-related characteristics according to age [37,38,39]. Previous results in healthy adults have shown positive associations between active commuting and well-being [40]. Women with fibromyalgia who spent more time walking without supervision showed a decrease in their perceived fatigue levels [6]. Taking a broader view, total PA has been associated with a reduction in the impact of fibromyalgia and fatigue scores in patients with fibromyalgia [13,41]. Such findings are in line with the current results for active commuting in the young group. Contrarily, the positive effects of PA on pain modulation observed previously [13,41,42,43] were not found in the current study for active commuting behaviors. Despite the association of active commuting in women with fibromyalgia with PA [11], this might not be sufficient enough PA to produce effects on pain modulation compared with the total PA. In addition to the PA duration, PA intensity plays an important role in pain modulation. A previous study has reported that fibromyalgia patients who increased their light PA improved or stabilized their pain levels, while those who increased their moderate PA increased their pain levels [44]. Active commuting might be a behavior that helps to modulate pain in women with fibromyalgia, but it is necessary to analyze this behavior in depth while controlling for the duration and intensity [45,46]. Therefore, there is a lack of studies that associate active commuting with symptoms experienced by patients with chronic pain or rheumatic conditions, making it difficult to understand whether the results obtained in this study are in accordance with previous evidence or were obtained by chance. Otherwise, active commuting showed similar results to those of previous studies found for symptomatology management through PA [4,5,6], except for pain modulation. This might suggest that active commuting interventions, as a source of PA, might help PA programs improve patients’ fibromyalgia-related characteristics.

Young and active commuters showed a lower fibromyalgia severity and a higher HRQoL than the young and passive group. However, the older and active commuter group showed similar fibromyalgia-related characteristics to the young and older passive groups. This difference between younger and older active commuters might be due to a decrease in PA with age. Even though active commuting remains stable after the age of approximately 35 in a healthy population [18], PA decreases with age [47], and women with fibromyalgia are less physically active than healthy women [48]. Although both groups usually walked to commute, the walking duration or the intensity might be different. However, there is a lack of data on the duration and intensity of active commuting, and we cannot explain with certainty the differences by age observed in the association between active commuting and fibromyalgia-related characteristics. The inclusion of Global Position Systems combined with accelerometry could be an appropriate technique that might cover this gap in future studies [45,46]. Accelerometry provides data on the intensity and duration of PA, and the use of Global Position Systems may help find locations at which populations are more active. These ideas may contribute to the promotion of PA in this population. Otherwise, recent studies suggest that patient perceptions’ of the negative effects of walking on symptomatology might negatively influence the inclusion of active commuting behaviors in their lifestyle [15,16]. Nevertheless, the current results did not display worse fibromyalgia-related characteristics for active commuters than passive commuters, regardless of the age group. Therefore, active commuting is a behavior that seems to produce benefits in fibromyalgia-related characteristics without adverse effects for women with fibromyalgia.

The results obtained indicate some better fibromyalgia-related characteristics in young women with fibromyalgia who commute actively, highlighting the importance of supporting this behavior as a source of PA. Studies that tested structured walking programs showed reductions in the symptoms of patients with fibromyalgia [49,50,51], but for a better adherence to these programs, specialist supervision is required [51]. Thus, active commuting strategies can be implemented in young women with fibromyalgia to attain health benefits through an increase in PA. In older women with fibromyalgia, other kinds of strategies (e.g., PA programs or unsupervised walking) might be more appropriate to obtain these health benefits. In the promotion of active commuting behaviors, different problems might appear, such as negative perceptions of the effect of walking on symptomatology [15,16] or difficulties in self-managing symptoms [52]. To overcome some of these anticipated negative consequences, support from the patients’ social environment (e.g., their family, friends, health providers, and fibromyalgia associations) is necessary [15]. Social networks for women with fibromyalgia might have a key role in the management of this illness, helping them to take part in activities and behaviors that have a direct effect on their fibromyalgia-related characteristics.

The cross-sectional design of the current study does not allow us to establish causal relationships. It seems plausible that active commuting might reduce some fibromyalgia-related characteristics, and it is equally possible that fibromyalgia-related characteristics have an effect on commuting behavior. Despite the reliability demonstrated by the commuting questionnaire [36], its validity is still unknown. Another limitation is the lack of data on the duration, intensity, and distance of commuting, which might be essential to understand the relationship between this behavior and fibromyalgia-related characteristics. Furthermore, we do not know whether these findings apply to men. A strength of the current study was the assessment of a relatively large sample size of women with fibromyalgia and our efforts to recruit a representative sample from Southern Spain [2]. Moreover, this is the first study that associates a specific PA domain such as active commuting with fibromyalgia-related characteristics.

## 5. Conclusions

We conclude that young women with fibromyalgia who actively commute are less impacted by fibromyalgia, have a higher HRQoL, and have lower fatigue scores than older and/or passive commuters. The possible positive effect of active commuting on fibromyalgia-related characteristics might decrease with age and with decreases in total PA. Physical activity programs for women with fibromyalgia focused on improving their fibromyalgia-related characteristics and health should consider active commuting behaviors.

## Figures and Tables

**Table 1:** Symptomatology characteristics and mode of commute in participants with fibromyalgia.

	All (*n* = 450)	Young Group (*n* = 194)	Older Group (*n* = 256)	*p*
	Mean (SD)	Mean (SD)	Mean (SD)	
Age	52.0 (8.0)	44.5 (4.4)	57.7 (4.7)	<0.001
Living with other household members (%) *	91.8/8.2	94.3/5.7	89.8/10.2	0.086
Educational level (%)				
No studies	10.7	4.1	15.6	<0.001
Primary school	48.2	49.0	47.7	0.783
Professional training	14.9	18.6	12.1	0.057
Secondary school	12.0	10.8	12.9	0.504
University degree	14.2	17.5	11.7	0.081
Current occupational status (%) ^‡^	26.0/74.0	35.0/65.0	19.1/80.9	<0.001
Body fat percentage	39.7 (8.9)	38.1 (7.9)	40.9 (9.5)	<0.001
Fibromyalgia severity (FIQR total score, 0–100)	64.5 (17.0)	63.2 (17.4)	65.5 (16.6)	0.150
Health-Related Quality of Life (SF36 physical score, 0–100)	29.6 (6.9)	29.9 (6.6)	29.5 (7.2)	0.529
Health-Related Quality of Life (SF36 mental score, 0–100)	35.6 (12.0)	35.7 (12.6)	35.6 (11.6)	0.947
Depressive symptoms (BDI-II, 0–36)	26.6 (11.5)	27.0 (12.3)	26.1 (10.9)	0.433
Sleep quality (PSQI total score, 0–21)	12.8 (3.9)	12.7 (4.1)	12.9 (3.6)	0.581
Pain (Pressure pain threshold, 0–8)	2.4 (0.8)	2.4 (0.7)	2.4 (0.8)	0.996
Pain (Pain catastrophizing total score, 0–52)	25.1 (12.7)	25.4 (12.8)	24.9 (12.7)	0.675
Pain (Chronic pain self-efficacy total score, 0–300)	135.9 (55.8)	142.6 (57.4)	130.9 (54.1)	0.027
Fatigue (MFI-S general fatigue, 4–20)	18.0 (2.6)	18.3 (2.4)	17.7 (2.6)	0.010
Active commuting, *n* (%)	310 (68.9)	133 (68.6)	177 (69.1)	0.895
To local shops, *n* (%)	342 (76.0)	152 (78.4)	190 (74.2)	0.309
To supermarkets, *n* (%)	208 (46.2)	79 (40.7)	129 (50.4)	0.042
To local services, *n* (%)	310 (68.9)	130 (67.0)	180 (70.3)	0.454

BDI-II, Beck Depression Inventory—Second Edition; FIQR, Revised Fibromyalgia Impact Questionnaire; MFI-S, Spanish version of the Multidimensional Fatigue Inventory; PSQI, Pittsburgh Sleep Quality Index; SD, standard deviation; SF36, 36-item Short-Form Health Survey. * Living with other household members is presented as living with others/living alone. ^‡^ Current occupational status is presented as working/studying or unemployed/retired. The sample size was *n* = 444 (*n* = 194 from the younger group and *n* = 250 from the older group). Between-group differences were tested by Student’s *t*-test for continuous variables and chi-square test for categorical variables.

**Table 2:** Comparison (linear regression analyses) of symptomatology characteristics between active and passive commuters from the younger (<51 years) and older (≥51 years) groups.

Young Group (<51 years)	Active Commuters	Passive Commuters	Difference	*p*
	*n* = 133	*n* = 61	Mean (95% CI)	
	Mean (SD)	Mean (SD)		
Fibromyalgia severity (FIQR—total score, 0–100)	60.71 (17.98)	68.49 (14.88)	7.605 (2.422 to 12.789)	0.004
Health-Related Quality of Life (SF36—physical score, 0–100)	31.00 (6.29)	27.46 (6.72)	−3.591 (−5.546 to −1.636)	0.001
Health-Related Quality of Life (SF36—mental score, 0–100)	36.29 (12.14)	34.27 (13.44)	−1.866 (−5.686 to 1.954)	0.338
Depressive symptoms (BDI-II, 0–36)	26.08 (11.90)	29.02 (13.01)	2.742 (−1.005 to 6.489)	0.143
Sleep quality (PSQI total score, 0–21)	12.31 (4.04)	13.43 (4.27)	1.131 (−0.117 to 2.378)	0.086
Pain (Pressure pain threshold, 0–8)	2.41 (0.72)	2.33 (0.75)	−0.079 (−0.303 to −0.143)	0.551
Pain (Pain catastrophizing total score, 0–52)	25.17 (13.10)	25.75 (12.19)	0.620 (−3.278 to 4.517)	0.782
Pain (Chronic pain self-efficacy total score, 0–300)	145.65 (59.20)	135.85 (53.15)	−9.423 (−26.814 to 7.968)	0.308
Fatigue (MFI-S general fatigue, 4–20)	17.99 (2.70)	18.98 (1.60)	0.999 (0.266 to 1.731)	0.013
**Older Group (≥51 years)**	***n* = 177**	***n* = 79**	**Mean (95% CI)**	
	**Mean (SD)**	**Mean (SD)**		
Fibromyalgia severity (FIQR—total score, 0–100)	64.99 (16.87)	66.57 (16.04)	1.734 (−2.661 to 6.129)	0.569
Health-Related Quality of Life (SF36—physical score, 0–100)	29.55 (7.42)	29.20 (6.61)	−0.292 (−2.199 to 1.614)	0.822
Health-Related Quality of Life (SF36—mental score, 0–100)	35.93 (11.43)	34.79 (11.90)	−1.253 (−4.332 to 1.825)	0.454
Depressive symptoms (BDI-II, 0–36)	25.80 (10.85)	26.92 (10.95)	1.013 (−1.887 to 3.913)	0.513
Sleep quality (PSQI total score, 0–21)	12.83 (3.67)	12.93 (3.60)	0.117 (−0.849 to 1.083)	0.855
Pain (Pressure pain threshold, 0–8)	2.38 (0.76)	2.42 (0.77)	0.044 (−0.158 to 0.246)	0.541
Pain (Pain catastrophizing total score, 0–52)	24.83 (12.75)	24.90 (12.54)	0.308 (−3.066 to 3.681)	0.956
Pain (Chronic pain self-efficacy total score, 0–300)	131.83 (53.98)	128.66 (54.68)	−2.828 (−17.162 to 11.507)	0.688
Fatigue (MFI-S general fatigue, 4–20)	17.69 (2.60)	17.65 (2.62)	–0.095 (−0.785 to 0.596)	0.725

BDI-II, Beck Depression Inventory—Second Edition; CI, confidence interval; FIQR, Revised Fibromyalgia Impact Questionnaire; MFI-S, Spanish version of the Multidimensional Fatigue Inventory; PSQI, Pittsburgh Sleep Quality Index; SD, standard deviation; SF36, 36-item Short-Form Health Survey.

## Data Availability

The data presented in this study are available upon request from the corresponding author due to ethical reasons.

## References

[1] Lee Y.C., Nassikas N.J., Clauw D.J. (2011). The role of the central nervous system in the generation and maintenance of chronic pain in rheumatoid arthritis, osteoarthritis and fibromyalgia. Arthritis Res. Ther..

[2] Segura-Jiménez V., Álvarez-Gallardo I.C., Carbonell-Baeza A., Aparicio V., Ortega F., Casimiro A., Delgado-Fernández M. (2015). Fibromyalgia Has a Larger Impact on Physical Health Than on Psychological Health, Yet Both are Markedly Affected: The Al-Ándalus Project. Semin. Arthritis Rheum..

[3] Skaer T.L., Kwong W.J. (2017). Illness perceptions and burden of disease in fibromyalgia. Expert Rev. Pharmacoecon. Outcomes Res..

[4] Geneen L., Smith B., Clarke C., Martin D., Colvin L.A., Moore R.A. (2014). Physical activity and exercise for chronic pain in adults: An overview of Cochrane reviews. Cochrane Database Syst. Rev..

[5] Segura-Jiménez V., Soriano-Maldonado A., Estévez-López F., Álvarez-Gallardo I.C., Delgado-Fernández M., Ruiz J.R., Aparicio V.A. (2017). Independent and joint associations of physical activity and fitness with fibromyalgia symptoms and severity: The al-Ándalus project. J. Sports Sci..

[6] Lopez-Roig S., Pastor M.-A., Penacoba C., Lledo A., Sanz Y., Velasco L. (2016). Prevalence and predictors of unsupervised walking and physical activity in a community population of women with fibromyalgia. Rheumatol. Int..

[7] Munguía-Izquierdo D., Legaz-Arrese A. (2012). Determinants of sleep quality in middle-aged women with fibromyalgia syndrome. J. Sleep Res..

[8] Gavilán-Carrera B., Delgado-Fernández M., Álvarez-Gallardo I.C., Acosta-Manzano P., Borges-Cosic M., Estévez-López F., Soriano-Maldonado A., Carbonell-Baeza A., Aparicio V.A., Segura-Jiménez V. (2023). Longitudinal association of sedentary time and physical activity with pain and quality of life in fibromyalgia. Scand. J. Med. Sci. Sport..

[9] Andrade A., Vilarino G.T., Sieczkowska S.M., Coimbra D.R., Bevilacqua G.G., Steffens R.d.A.K. (2020). The relationship between sleep quality and fibromyalgia symptoms. J. Health Psychol..

[10] Borges-Cosic M., Aparicio V.A., Estévez-López F., Soriano-Maldonado A., Acosta-Manzano P., Gavilán-Carrera B., Delgado-Fernández M., Geenen R., Segura-Jiménez V. (2019). Sedentary time, physical activity, and sleep quality in fibromyalgia: The al-Ándalus project. Scand. J. Med. Sci. Sport..

[11] Herrador-Colmenero M., Segura-Jiménez V., Álvarez-Gallardo I.C., Soriano-Maldonado A., Camiletti-Moirón D., Delgado-Fernández M., Chillón P. (2022). Is active commuting associated with sedentary behaviour and physical activity in women with fibromyalgia? The al-Ándalus project. Disabil. Rehabil..

[12] Mueller N., Rojas-Rueda D., Cole-Hunter T., de Nazelle A., Dons E., Gerike R., Götschi T., Int Panis L., Kahlmeier S., Nieuwenhuijsen M. (2015). Health impact assessment of active transportation: A systematic review. Prev. Med..

[13] Kaleth A.S., Slaven J.E., Ang D.C. (2014). Increasing Steps/Day Predicts Improvement in Physical Function and Pain Interference in Adults with Fibromyalgia. Arthritis Care Res..

[14] López-Pousa S., Bassets Pagès G., Monserrat-Vila S., De Gracia Blanco M., Hidalgo Colomé J., Garre-Olmo J. (2015). Sense of Well-Being in Patients with Fibromyalgia: Aerobic Exercise Program in a Mature Forest—A Pilot Study. Evid.-Based Complement. Altern. Med..

[15] Sanz-Baños Y., Pastor M.Á., Velasco L., López-Roig S., Peñacoba C., Lledo A., Rodríguez C. (2016). To walk or not to walk: Insights from a qualitative description study with women suffering from fibromyalgia. Rheumatol. Int..

[16] Peñacoba C., Pastor M.-Á., López-Roig S., Velasco L., Lledo A. (2016). Walking Beliefs in Women with Fibromyalgia: Clinical Profile and Impact on Walking Behavior. Clin. Nurs. Res..

[17] Herrador-Colmenero M., Álvarez-Gallardo I.C., Segura-Jiménez V., Estévez-López F., Soriano-Maldonado A., Ruiz-Montero P.J., Girela-Rejón M.J., Delgado-Fernández M., Chillón P. (2016). Associations between patterns of active commuting and socioeconomic factors in women with fibromyalgia: The al-Ándalus Project. Clin. Exp. Rheumatol..

[18] Menai M., Charreire H., Feuillet T., Salze P., Weber C., Enaux C., Andreeva V.A., Hercberg S., Nazare J., Perchoux C. (2015). Walking and cycling for commuting, leisure and errands: Relations with individual characteristics and leisure-time physical activity in a cross-sectional survey (the ACTI-Cités project). Int. J. Behav. Nutr. Phys. Act..

[19] Segura-Jiménez V., Estévez-López F., Soriano-Maldonado A., Álvarez-Gallardo I., Delgado-Fernández M., Ruiz J., Aparicio V. (2016). Gender differences in symptoms, health-related quality of life, sleep quality, mental health, cognitive performance, pain-cognition, and positive health in Spanish fibromyalgia individuals: The Al-Ándalus project. Pain Res. Manag..

[20] Ghavidel-Parsa B., Bidari A., Maafi A.A., Ghalebaghi B. (2015). The Iceberg nature of fibromyalgia burden: The clinical and economic aspects. Korean J. Pain.

[21] Macfarlane G.J., Kronisch C., Dean L.E., Atzeni F., Häuser W., Fluß E., Choy E., Kosek E., Amris K., Branco J. (2017). EULAR revised recommendations for the management of fibromyalgia. Ann. Rheum. Dis..

[22] Estévez-López F., Segura-Jiménez V., Álvarez-Gallardo I.C., Borges-Cosic M., Pulido-Martos M., Carbonell-Baeza A., Aparicio V.A., Geenen R., Delgado-Fernández M. (2017). Adaptation profiles comprising objective and subjective measures in fibromyalgia: The al-Ándalus project. Rheumatology.

[23] Wolfe F., Smythe H.A., Yunus M.B., Bennett R.M., Bombardier C., Goldenberg D.L., Tugwell P., Campbell S.M., Abeles M., Clark P. (1990). The American College of Rheumatology 1990 criteria for the classification of fibromyalgia. Arthritis Rheum..

[24] Folstein M.F., Folstein S.E., McHugh P.R. (1975). “Mini-mental state”. A practical method for grading the cognitive state of patients for the clinician. J. Psychiatr. Res..

[25] Malavolti M., Mussi C., Poli M., Fantuzzi A., Salvioli G., Battistini N., Bedogni G. (2003). Cross-calibration of eight-polar bioelectrical impedance analysis versus dual-energy X-ray absorptiometry for the assessment of total and appendicular body composition in healthy subjects aged 21–82 years. Ann. Hum. Biol..

[26] Segura-Jiménez V., Aparicio V., Álvarez-Gallardo I., Carbonell-Baeza A., Tornero-Quiñones I., Delgado-Fernández M. (2015). Does body composition differ between fibromyalgia patients and controls? The al-Ándalus project. Clin. Exp. Rheumatol..

[27] Bennett R.M., Friend R., Jones K.D., Ward R., Han B.K., Ross R.L. (2009). The Revised Fibromyalgia Impact Questionnaire (FIQR): Validation and psychometric properties. Arthritis Res. Ther..

[28] Alonso J., Prieto L., Antó J. (1995). The Spanish version of the SF-36 Health Survey (the SF-36 health questionnaire): An instrument for measuring clinical results. Med. Clin..

[29] Wiebe J.S., Penley J.A. (2005). A psychometric comparison of the Beck Depression Inventory-II in English and Spanish. Psychol. Assess..

[30] Beck A., Steer R., Brown G. (1996). Manual for the Beck Depression Inventory-II.

[31] Macías J.A., Royuela A. (1996). The Spanish version of the Pittsburg Sleep Quality Index. Inf. Psiquiátricas.

[32] García J., Rodero B., Alda M., Sobradiel N. (2008). Validation of the Spanish version of the Pain Catastrophizing Scale in fibromyalgia. Med. Clin..

[33] Martin-Aragon M., Pastor M., Rodriguez-Marin J., March M., Lledo A., Lopez-Roig S., Terol M. (1999). Self-efficacy perception in chronic pain: Adaptation and validation of the Chronic Pain Self-Efficacy Scale. Rev. Psicol. Salud.

[34] Schwarz R., Krauss O., Hinz A. (2003). Fatigue in the general population. Onkologie.

[35] Munguía-Izquierdo D., Segura-Jiménez V., Camiletti-Moirón D., Pulido-Martos M., Álvarez-Gallardo I., Romero A., Aparicio V., Carbonell-Baeza A., Delgado-Fernández M. (2012). Multidimensional Fatigue Inventory: Spanish adaptation and psychometric properties for fibromyalgia patients. The Al-Andalus study. Clin. Exp. Rheumatol..

[36] Herrador-Colmenero M., Ruiz J.R., Ortega F.B., Segura-Jiménez V., Alvarez-Gallardo I.C., Camiletti-Moirón D., Estévez-López F., Delgado-Fernández M., Chillón P. (2015). Reliability of the ALPHA environmental questionnaire and its association with physical activity in female fibromyalgia patients: The al-Ándalus project. J. Sports Sci..

[37] Ubago Linares M.d.C., Ruiz-Pérez I., Bermejo Pérez M.J., Olry de Labry-Lima A., Hernández-Torres E., Plazaola-Castaño J. (2008). Analysis of the impact of fibromyalgia on quality of life: Associated factors. Clin. Rheumatol..

[38] Jiao J., Vincent A., Cha S.S., Luedtke C.A., Oh T.H. (2014). Relation of age with symptom severity and quality of life in patients with fibromyalgia. Mayo Clin. Proc..

[39] Cronan T., Serber E., Walen H., Jaffe M. (2002). The influence of age on consumption. J. Aging Health.

[40] Martin A., Goryakin Y., Suhrcke M. (2014). Does active commuting improve psychological wellbeing? Longitudinal evidence from eighteen waves of the British Household Panel Survey. Prev. Med..

[41] Segura-Jiménez V., Borges-Cosic M., Soriano-Maldonado A., Estévez-López F., Álvarez-Gallardo I.C., Herrador-Colmenero M., Delgado-Fernández M., Ruiz J.R. (2017). Association of sedentary time and physical activity with pain, fatigue, and impact of fibromyalgia: The al-Ándalus study. Scand. J. Med. Sci. Sports.

[42] Jones K.D., Adams D., Winters-Stone K., Burckhardt C.S. (2006). A comprehensive review of 46 exercise treatment studies in fibromyalgia (1988–2005). Health Qual. Life Outcomes.

[43] McLoughlin M., Stegner A., Cook D. (2012). The relationship between physical activity and brain responses to pain in fibromyalgia. J. Pain.

[44] Jenssen M.D.K., Salvi E., Fors E.A., Nilsen O.A., Ngo P.D., Tejedor M., Bellika J.G., Godtliebsen F. (2024). Exploring Pain Reduction through Physical Activity: A Case Study of Seven Fibromyalgia Patients. Bioengineering.

[45] Remmers T., Van Kann D., Kremers S., Ettema D., De Vries S.I., Vos S., Thijs C. (2020). Investigating longitudinal context-specific physical activity patterns in transition from primary to secondary school using accelerometers, GPS, and GIS. Int. J. Behav. Nutr. Phys. Act..

[46] Liu W., Chambers T., Clevenger K.A., Pfeiffer K.A., Rzotkiewicz Z., Park H., Pearson A.L. (2024). Quantifying time spent outdoors: A versatile method using any type of global positioning system (GPS) and accelerometer devices. PLoS ONE.

[47] Hallal P.C., Andersen L.B., Bull F.C., Guthold R., Haskell W., Ekelund U., Alkandari J.R., Bauman A.E., Blair S.N., Brownson R.C. (2012). Global physical activity levels: Surveillance progress, pitfalls, and prospects. Lancet.

[48] Segura-Jiménez V., Álvarez-Gallardo I., Estévez-López F., Soriano-Maldonado A., Delgado-Fernández M., Ortega F.B., Aparicio V.A., Carbonell-Baeza A., Mota J., Silva P. (2015). Differences in sedentary time and physical activity between women with fibromyalgia and healthy controls: The al-Ándalus project. Arthritis Rheumatol..

[49] Ecija C., Catala P., Velasco L., Pastor-Mira M.A., Peñacoba C. (2022). When It Hurts, a Positive Attitude May Help. The Moderating Effect of Positive Affect on the Relationship Between Walking, Depression, and Symptoms in Women with Fibromyalgia. Pain Manag. Nurs..

[50] Sanromán L., Catalá P., Écija C., Suso-Ribera C., Román J.S., Peñacoba C. (2022). The Role of Walking in the Relationship between Catastrophizing and Fatigue in Women with Fibromyalgia. Int. J. Environ. Res. Public Health.

[51] Sanz-Baños Y., Pastor-Mira M.Á., Lledó A., López-Roig S., Peñacoba C., Sánchez-Meca J. (2018). Do women with fibromyalgia adhere to walking for exercise programs to improve their health? Systematic review and meta-analysis. Disabil. Rehabil..

[52] Jerant A.F., Von Friederichs-Fitzwater M.M., Moore M. (2005). Patients’ perceived barriers to active self-management of chronic conditions. Patient Educ. Couns..

